# Prevalence of gastrointestinal bleeding and frequency of selected predictors of mortality on the medical emergency ward at Mulago hospital

**DOI:** 10.4314/ahs.v23i1.66

**Published:** 2023-03

**Authors:** Ivan Kisuule, Emmanuel Seremba, Magid Kagimu

**Affiliations:** 1 Gastroenterology Division, Department of Medicine, Makerere University College of Health Sciences, Kampala, Uganda; 2 Mulago National Referral Hospital, Kampala, Uganda

**Keywords:** Gastrointestinal bleeding, predictors of mortality, Emergency ward

## Abstract

**Background:**

There was no data on the prevalence of Gastrointestinal bleeding (GI) among patients admitted on the emergency ward at Mulago hospital. This was partly because the medical records were not adequately completed as designed.

**Objective:**

To estimate the prevalence of gastrointestinal bleeding and the frequency of selected predictors of mortality on the emergency ward.

**Methods:**

This was a chart review incorporating quality improvement methods in the process of data collection. The health care team was educated on documentation of gastrointestinal bleeding while being assessed weekly for knowledge and practice of completion of the Casualty Assessment form (CAF) from which a documented diagnosis of GI bleeding and selected predictors of mortality were looked for.

**Results:**

Of the 1881 CAF assessed, 278 had a documented diagnosis of GI bleeding, resulting in a prevalence of 6.8%. Of the patients with GI bleeding, 14.1% had age greater than 60 years, 24.0% had a systolic blood pressure less than 100mmHg and 44.5% had a heart rate greater than 100 beats per minute.

**Conclusion:**

The prevalence of GI bleeding on the medical emergency ward of Mulago hospital is high. This calls for strategies for resuscitative management of this life-threatening medical emergency. Among the selected predictors of mortality, tachycardia was most frequent followed by hypotension. These should always be assessed in a patient with GI bleeding and resuscitative measures with blood transfusion and intravenous fluids undertaken to correct them.

## Background

Gastrointestinal bleeding is classified as upper or lower gastrointestinal bleeding, separated anatomically by the ligament of Treitz. Acute upper gastrointestinal bleeding should be suspected in patients with haematemesis, coffee-ground vomiting, melaena or unexplained fall in haemoglobin. In up to 20% of cases, acute upper gastrointestinal bleeding (AUGIB) may mimic lower gastrointestinal bleeding. Features that predict AUGIB in cases of haematochezia include haemodynamic instability, increased serum urea: creatinine ratio, and reduced haematocrit. The diagnosis is confirmed with endoscopy, which may also serve to provide therapeutic interventions^1^.

Acute lower gastrointestinal bleeding manifests as hematochezia (maroon or red blood passed through the rectum). Uncommonly, lower gastrointestinal bleeding can manifest as melena (black, tarry stools), or, conversely, brisk (rapid) upper gastrointestinal bleeding can manifest as hematochezia^2^.

At the initial encounter with a patient with gastrointestinal bleeding, risk assessment is performed to determine the severity of upper gastrointestinal bleeding according to vital signs and patient factors. Tachycardia (heart rate ≥100 beats per minute), hypotension (systolic blood pressure ≤100 mm Hg), age older than 60 years, and major coexisting conditions are associated with an increased risk of further bleeding and death^3^.

Risk-assessment tools are available and are useful in identifying patients at risk of mortality. For example, discharge from the emergency department followed by outpatient care has been suggested for patients with a Glasgow-Blatchford score of 0, 0 to 1, or, in patients who are less than 70 years of age, 0 to 2 (on a scale of 0 to 23, with higher scores indicating higher risk). A prospective study showed that when hospitalized; less than 1% of such patients require intervention and less than 0.5% die^4^.

There are no current data on the prevalence of GI bleeding and selected predictors of mortality on the medical emergency ward. This is partly because the medical records are not adequately completed as designed. For example, the casualty assessment form was designed to be completed by the health care team and left in the emergency ward after the patient is either discharged or transferred to another ward. Reviewing data from this form if it is adequately completed and left on the emergency ward would provide data that can be used to estimate the prevalence of various conditions. Unfortunately, this form is neither regularly adequately completed, nor is it left on the emergency ward in many instances when the patient is being transferred for specialized care. This poor clinical documentation practice in our setting had also been recognized among other challenges that affect optimal care of severely ill patients in resource limited settings^5^. To obtain a more accurate estimate of the prevalence of GI bleeding on the emergency ward it was essential to improve the process of documentation and storage of the data on the casualty assessment form by using quality improvement methods.

A more accurate estimation of the prevalence of gastrointestinal bleeding on the emergency ward of Mulago hospital would inform policy on the burden of this condition and lead to better planning for strategies and resources required to care for these patients such as blood for blood transfusion, drugs like terlipressin and space for resuscitation of these patients. Since the current prevalence of GI bleeding on the emergency ward was not known, proper planning for improving the care of these patients was unlikely to be done and some of these patients whose lives could have been saved with adequate resuscitation, might die.

The general objective was to determine the prevalence of GI bleeding and selected predictors of mortality among patients admitted on the medical emergency ward of Mulago hospital, to generate more accurate information to contribute to improving patient care by using it in planning strategies for adequate resuscitation of these patients.

## Methods

### Study Design and Setting

This was a chart review incorporating quality improvement methods in the process of data collection. The study was conducted on the medical emergency ward of the Directorate of Medicine, Mulago hospital, which was operating in Kiruddu, Kampala, Uganda. This emergency ward has a bed capacity of 25 beds and receives an average of 30 patients in a 24-hour period and 900 patients over a month period. The patients stay in the emergency ward for a short period after which they are either admitted to the specialized ward with respect to their disease or are discharged home if they do not need to be admitted. The emergency healthcare team is composed of eight medical officers, fifteen nurses, four intern doctors and one physician. These were the members of the health team we primarily targeted to get involved in the study.

The study was conducted between July and October 2018. We used quality improvement methods in the process of data collection so that we could estimate the prevalence of GI bleeding on the emergency ward of Mulago hospital with more accuracy. The QI methods included mapping of the documentation process, identifying the root causes of inadequate documentation through focus group discussions and key informant interviews, intervening by educating health workers on the emergency ward regarding documentation on the CAF and then assessed the trend of completion of the form while looking out for the documented diagnosis of GI bleeding and the selected predictors of mortality namely age, systolic blood pressure and pulse rate.

### Data Sources

The data were collected using a data collection tool by the principal investigator and research assistants who first conducted the focus group discussions and key informant interviews to find out the root causes of inadequate completion of the CAF and then did education sessions with ongoing knowledge and CAF completion assessment. We used the Plan-Do-Study-Act quality improvement method during the data collection process where we planned and did interventions (education and supervision of health workers), studied how they responded to filling of the CAF and acted by continuous education and supervision of health workers.

### Sample size and sampling method

Since there was a component of improving documentation of medical records for all patients attending the emergency ward during the study period of 13 weeks, we studied the casualty assessment forms of all these patients. Therefore, our final sample size was determined by the number of casualty assessment forms that were collected over a period of 13 weeks. There was no interaction with the patients but their casualty assessment forms which had already been filled by the attending health care team were studied. Consecutive sampling of the casualty assessment forms which were filled by the quality improvement team during the study period of 13 weeks was done.

### Data analysis and management

Data collected were entered into the computer using EPI-DATA (version 3.1) software to minimize data entry errors and exported to STATA/IC 11.0 for analysis. Data were then backed up and archived in both soft and hard copies to avoid loss. Confidentiality was ensured by de-identification using numbers instead of patients' names. Qualitative data were recorded by the notes taker and then transcribed into themes to aid in qualitative data analysis. Quantitative data on selected predictors of mortality that is; age, systolic blood pressure and pulse rate were analyzed using descriptive statistics.

### Ethical considerations

Approval to conduct the study was sought from the Department of Medicine, Makerere University College of Health Sciences and from the School of Medicine Research and Ethics Committee (SOM-REC).

## Results

### Social-demographic characteristics of the study participants

The social-demographic characteristics of the study participants who had a documented diagnosis of GI bleeding are summarized in table 1 below. Among those with a diagnosis of GI bleeding, majority were male 77 (60.2%), of Catholic faith 44 (34.4%) and from central Uganda 60 (46.9%).

**Table 1:** Demographic Characteristics of patients with GI bleeding.

Characteristic	Frequency (N=128)	Percentage
**Age (mean, SD)**	41.3, 17.2	
**Sex**		
Male	77	60.2
Female	51	39.8
**Religion**		
Muslim	18	14.1
Catholic	44	34.4
Anglican	3	2.3
Pentecostal	35	27.3
Others	19	14.8
Not recorded	9	7.0
**Occupation**		
Employed	49	38.3
Not employed	43	33.6
Others	8	6.3
Not recorded	28	21.9
**Region of origin in Uganda**		
Central	60	46.9
West	31	24.2
East	16	12.5
North	9	7.0
Not recorded	12	9.4
**Outcome on transfer for specialised care**		
Improved	5	3.9
Unimproved	121	94.5
Died	2	1.6

### Average percentage completion of the CAF

**Figure 2:**
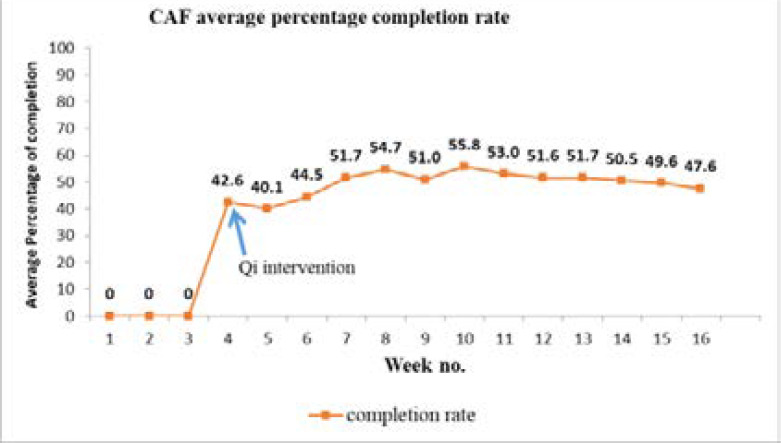
Average percentage completion of the fields of the CAF

The average weekly percentage completion was the proportion of the fields on the CAF that were filled. For example, in week four, when the interventions (education and supervision of health workers) were introduced, the average percentage of completion of the 40 CAF fields in that week was 42.6%. In the first three weeks before intervention the casualty assessment forms were not being filled and also not left behind in the emergency ward when the patient was either discharged home or sent to another ward for further management and therefore the completion rate in that period was zero. The run chart in figure 2 below demonstrates the trend in the average percentage completion of the CAF.

### Completion of diagnosis field of the CAF

**Figure 3:**
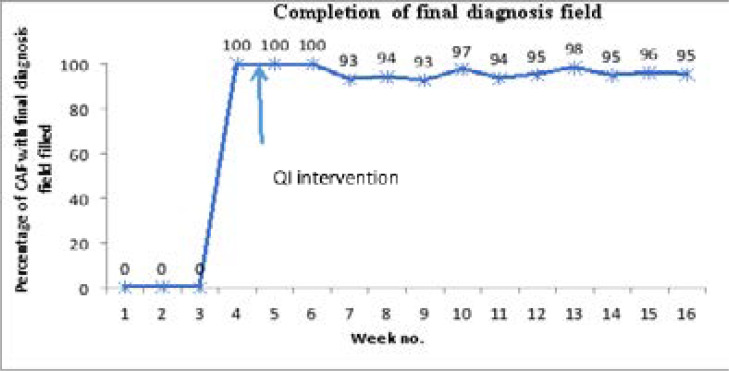
Run chart showing the average percentage completion of the final diagnosis field on the CAF among all patients

Among the fields on the CAF is the diagnosis field where the patient's suspected or confirmed disease is written and determines the specialized ward to which the patient should be spent. It also helps to collect data on the prevalence of different diseases on the emergency ward, of which GI bleeding was our disease of interest. We therefore assessed for the completion of this field since it was our source of data for determining the prevalence of GI bleeding on the emergency ward during the study period. The run chart in figure 3 below shows the average percentage completion of the final diagnosis field on the CAF for all study patients.

### Prevalence of gastrointestinal bleeding

**Figure 4:**
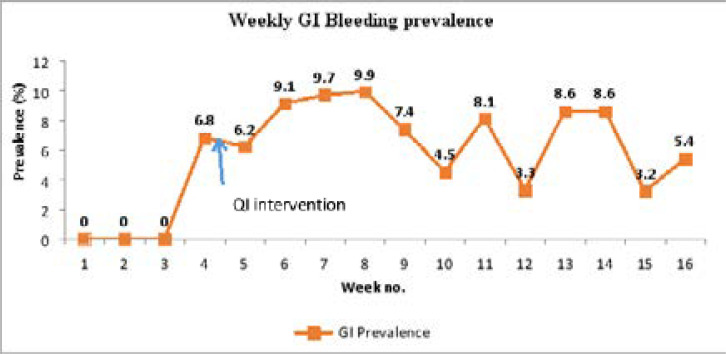
Run chart showing weekly prevalence of GI bleeding

Out of the 1881 CAFs assessed during the study period, 128 of these forms had a documented diagnosis of gastrointestinal bleeding, giving an overall prevalence of 6.8%. The weekly prevalence ranged between 3.2% and 9.8%. These findings are presented on the run chart in figure 4 below.

### Predictors of mortality among patients with gastrointestinal bleeding

The selected clinical characteristics which predict a high risk of mortality among patients with GI bleeding were age, systolic blood pressure and pulse rate. These predictors were selected based on the Rockall score which is a validated score as presented in the background. The median age of patients with GI bleeding was 39 years with interquartile range of 13 to 80 years. Only 18 (14.1%) patients were greater than 60 years of age. Hypotension (systolic blood pressure less than 100mmHg) was observed in 30 (24.0%) patients while tachycardia (pulse rate greater than 100 beats per minute) was recorded for 57 (44.5%) of the patients. Further, 15 (11.7%) of the patients had both hypotension and tachycardia. Only one patient had age greater than 60 years, hypotension, and tachycardia. Two patients died on the emergency ward and both had hypotension but were less than 60 years and had no tacycardia. The table below summarizes these findings.

**Table 2:** Proportion of GI bleeding patients with selected predictors of mortality

Clinical Parameter	Number of GI Bleeding patients (N=128)	Proportion
Age >60yrs	18	14.1%
SBP<100mmHg	30	24.0%
HR>100bpm	57	44.5%

NB: Percentages not mutually exclusive

## Discussion

This study aimed to determine the prevalence of gastrointestinal bleeding and the frequency of selected predictors of mortality on the medical emergency ward of Mulago hospital with quality improvement methods incorporated in the process of data collection from the casualty assessment forms (CAF). Before the study was done, the CAF were not being filled adequately and were not being kept on the emergency ward and therefore would not be used to give us an estimate of the prevalence of GI bleeding. We therefore used quality improvement methods to improve the process of documentation on the CAF so that we estimate the prevalence of GI bleeding with more accuracy. The prevalence of GI bleeding obtained after improving the documentation process is more accurate and reflects the burden of this life-threatening medical emergency.

We found a prevalence of gastrointestinal bleeding of 6.8%. To our knowledge, this is the first study in Uganda to study the prevalence of GI bleeding. This prevalence is higher than the prevalence of 2.0% that was found in a Nigerian study^6^. However, this study only looked at upper gastrointestinal bleeding at the emergency department of a tertiary hospital in Nigeria and much as it was a retrospective study based on review of medical records, they did not incorporate quality improvement methods in the process of data collection. Indeed, they stated missing data as a major limitation in their study. Other studies in similar resource setting have evaluated causes of GI bleeding^7^–^9^ and as such, it is not possible to compare our findings with theirs since we did not study etiology of the GI bleeding in our study. Gastrointestinal bleeding has been characterized better in high resource settings with incidence rates of 134 per 100,000 in the UK^1^ and being the most common cause of hospitalization due to gastrointestinal disease in the United States, accounting for more than 507,000 hospitalizations annually in the US^10^. Basing on the Rockall criteria^3^, our patients were at a low risk of mortality. This is attributed to the fact that only 14.1% were above the age of 60 years; hypotension was prevalent in only 24% and tachycardia in 44.5%. These findings add support to earlier findings that mortality from GI bleeding is largely the result of complications associated with other illnesses rather than bleeding to death^11^.

## Strength of the study

We incorporated quality improvement methods in the process of data collection which improved the quality of data generated and therefore estimated the prevalence of GI bleeding and selected predictors of mortality with more accuracy.

## Limitations of the study

We abstracted the diagnosis of GI bleeding as recorded on the CAF. These were not confirmed by endoscopy. However, the diagnosis of GI bleeding can be made accurately from the clinical presentation without endoscopy. Even with quality improvement measures, the CAF were not completed adequately. However, the estimated prevalence may not be far from the true prevalence since the diagnosis field was well completed during the study period.

## Conclusions

The prevalence of GI bleeding on the medical emergency ward of Mulago hospital is higher than other resource limited settings with 1 in every 15 patients having this condition. This calls for strategies for resuscitative management of this potentially life-threatening medical emergency. Among the selected predictors of mortality, tachycardia was most frequent followed by hypotension. These should always be assessed in a patient with GI bleeding and resuscitative measures with blood transfusion and intravenous fluids undertaken to correct them. Further research is needed to establish the causes of GI bleeding in our setting and measures needed to prevent them.

## Recommendations

Basing on the prevalence of GI bleeding on the emergency ward, there is need for creation of triage and resuscitation areas to allow for quick identification and effective emergency care of these patients. In addition, blood, and other lifesaving medications such as terlipressin and proton pump inhibitors should be availed for this medical emergency. Continuous education and supervision of health workers should be done to sustain proper completion of the CAF.

## Figures and Tables

**Figure 1:**
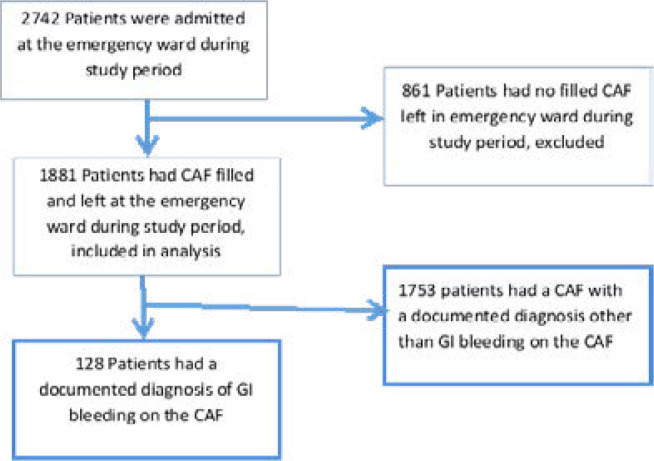
Study flow diagram

## Data Availability

The datasets used or analysed during this study are available from the corresponding author on reasonable request.

## Abbreviations

CAF: Casualty assessment form
GI: Gastrointestinal
HR: Heart rate
QI: Quality improvement.
SBP: Systolic blood pressure
